# Low Temperature Soda-Oxygen Pulping of Bagasse

**DOI:** 10.3390/molecules21010085

**Published:** 2016-01-13

**Authors:** Fengxia Yue, Ke-Li Chen, Fachuang Lu

**Affiliations:** 1 Faculty of Chemical Engineering, Kunming University of Science and Technology, Kunming 650500, China; fyue@wisc.edu; 2 State Kay Laboratory of Pulp and Paper Engineering, South China University of Technology, Guangzhou 510640, China; 3 Department of Biochemistry and Great Lakes Bioenergy Research Center, University of Wisconsin-Madison, Madison, WI 53726, USA

**Keywords:** bagasse, brightness, Kappa number, low temperature, soda-oxygen pulping, environmentally friendly, energy-saving

## Abstract

Wood shortages, environmental pollution and high energy consumption remain major obstacles hindering the development of today’s pulp and paper industry. Energy-saving and environmental friendly pulping processes are still needed, especially for non-woody materials. In this study, soda-oxygen pulping of bagasse was investigated and a successful soda-oxygen pulping process for bagasse at 100 °C was established. The pulping parameters of choice were under active alkali charge of 23%, maximum cooking temperature 100 °C, time hold at maximum temperature 180 min, initial pressure of oxygen 0.6 MPa, MgSO_4_ charge 0.5%, and de-pithed bagasse consistency 12%. Properties of the resultant pulp were screened yield 60.9%, Kappa number 14, viscosity 766 dm^3^/kg, and brightness 63.7% ISO. Similar pulps were also obtained at 110 °C or 105 °C with a cooking time of 90 min. Compared with pulps obtained at higher temperatures (115–125 °C), this pulp had higher screened yield, brightness, and acceptable viscosity, while the delignification degree was moderate. These results indicated that soda-oxygen pulping at 100 °C, the lowest cooking temperature reported so far for soda-oxygen pulping, is a suitable process for making chemical pulp from bagasse. Pulping at lower temperature and using oxygen make it an environmental friendly and energy-saving pulping process.

## 1. Introduction

The pulp and paper industry has been playing an increasingly important role in the development of the modern economy as paper and board consumption continuously increase across the world [1]. However, the available wood supply in many countries cannot meet the growing demand [2]. This demand for paper must be reconciled with growing environmental concerns. Enhancing energy efficiency and reducing the consumption of wood are critical for the pulp and paper industry because of their impact on competitiveness and the environmental implications. Therefore, there is a need to increase the use of non-wood fibers (e.g., previously unexplored annual or perennial plant species, agriculture and forest residues) and to develop more efficient, less polluting pulping process for the paper industry. The primary interest in non-wood materials is due to the fact that they provide fibers of excellent quality for making specialty papers or constitute the sole affordable source of fibrous raw materials in some geographical areas where non-wood plants are an alternative to the increasingly scant forest wood for pulp fibers [3]. In China, the use of non-wood fibers has played an important role in the paper industry for decades. Bagasse fiber, a residual by-product obtained after the extraction of juice from the crushed stalks of sugar cane, is one of the most important renewable raw materials for the papermaking industry in China. There are also many international studies on bagasse pulping [4,5,6]. Pulping of bagasse has been carried out mostly using the soda, sulfate and neutral sulfite methods [7]. However, there are still many problems in the practice of bagasse pulp production, such as high cooking temperatures, low pulp yields, troublesome environmental pollution, and so on.

The soda-oxygen bleaching method was developed in 1961 and has been commercialized since the beginning of the 1970s as a way to minimize environmental pollution. As a pulping technology, it has some advantages over the conventional soda pulping processes due to the presence of oxygen that helps delignification of fibrous raw materials and reduces the chloronome groups in the resultant pulps. In addition, this pulping method is particularly adequate for low-density raw materials [8] including most non-wood fibers (e.g., wheat straw, bagasse) [9]. Compared with the soda-anthraquinone and soda processes, soda-oxygen pulping gives the highest retention of silica in the pulp, and thus the least amount of silica left in the spent pulping liquor. Therefore, the soda-oxygen pulping method offers a highly practical and efficient solution to the silica problem associated with non-wood pulping [10]. Furthermore, the black liquor from such non-wood pulping process has the potential of being recycled without any further treatment (we have tested recycling the spent liquid at least five times before discharging without any problem), so soda-oxygen pulping technology can be considered as a cleaner pulping process according to the “cleaner production” standard [11,12]. One of the obvious advantages for soda-oxygen pulping is its much lower cooking temperature, thus using less energy, over the conventional chemical pulping while providing pulps with comparable properties. Nevertheless, the temperature of 120 °C or higher, commonly applied in soda-oxygen pulping, could still inevitably result in significant degradation of the pulp. Using a lower cooking temperature for pulping would be of great benefit to pulp production and the commercialization of soda-oxygen pulping as well. In this study, we explored the potential of soda-oxygen pulping performed at even lower temperatures with the aim of establishing a clean, energy-saving pulping system suitable for non-wood materials.

## 2. Results and Discussion

### 2.1. Chemical Composition of Depithed Bagasse

The de-pithed bagasse used in this study contained 77.1% holocellulose, 20.2% lignin, and 48.8% cellulose (Table 1). The de-pithed bagasse from Xinping is somewhat different in chemical composition from that from other geographical locations. This can be traced back to differences during the harvesting, storage, de-pithing, and location, as well as in the habitats of the raw materials employed [4]. Compared with the composition of other agricultural residues, bagasse has higher content of cellulose, relatively lower content of lignin (Table 1), and much lower silica ash than rice straw and wheat straw. Therefore, bagasse pulp could produce paper having better properties for writing and printing [4,7,13,14,15]. The content of 1% NaOH extractives in bagasse used for this study was high, but in the low range as reported, which indicated easy access and dissolution of the cell wall components by low or moderate alkali charge [4,13]. Compared to woody materials, bagasse should be pulped more easily due to its lower lignin content (20.2%). Thus a low to moderate alkali charge could be used in order to preserve the cellulose and pentosans. The corresponding high pentosans content (27.6%) of bagasse, if preserved in pulp during the pulping process, could produce pulps with higher yields. In addition, it was predicted from the favorable cellulose-to-lignin ratio that soda-oxygen pulping would give the bagasse used in this study a higher yield.

**Table 1:** Chemical composition of de-pithed bagasse [4,16] and other non-wood materials [7,14,15].

Species	Holecellulose (%)	Cellulose ^a^ (%)	Klason Lignin (%)	Pentosans (%)	1% NaOH Extractive (%)	Cellulose: Lignin
Bagasse (Xinping)	77.1	48.8	20.2	24.3	28.5	2.4
Bagasse (refs)	ND ^b^	46.2–54.8	17.6–22.9	23.0–27.6	27.7–41.0	2.0–3.1
Wheat straw	ND ^b^	38.2	15.1	ND ^b^	ND ^b^	2.5
Rice straw	58.9	34.4	22.9	ND ^b^	ND ^b^	1.5
*Carpolobia lutea*	ND ^b^	44.1	26.0	ND ^b^	21.0	1.7

^a^ Determined by a mixture of nitric acid-ethanol; ^b^ Not determined.

### 2.2. Soda-Oxygen Pulping of Bagasse at Lower Temperatures

Soda-oxygen pulping involves complicated heterogeneous reactions with multiple gas–liquid–solid phases. The delignification in this process is a synergistic result of the action of alkali and oxygen on the components of the raw materials. In order to minimize the degradation of carbohydrates caused by alkali and oxygen at high temperature, the cooking temperature of soda-oxygen pulping is usually lower (around 130 °C) than that of traditional chemical pulping [8,17]. Furthermore, the relative low selectivity of oxygen delignification also leads to significant degradation of carbohydrates during cooking. However, these drawbacks of oxygen delignification could be overcome by adding suitable chemicals called carbohydrate protectors to inhibit carbohydrate degradation. It is a common practice for soda-oxygen pulping to add protective agents in addition to alkali and oxygen. To date, the magnesium compounds, such as MgSO_4_, are the best and most widely used reagents to improve the selectivity of oxygen delignification [18,19]. Therefore, MgSO_4_ was used as an additive to protect the carbohydrates during the pulping process of this study.

The selectivity of soda-oxygen pulping could be considered as the ratio of the rate of delignification to the rate of carbohydrate degradation. It is more common to evaluate a cooking condition by convenient measures such as Kappa number of pulp instead of lignin content, the viscosity of pulp instead of cellulose degradation [20]. In general, yield, Kappa number, and viscosity are key criteria for the evaluation of pulp qualities dependent upon the cooking conditions. For soda-oxygen pulping the brightness of pulp is also considered. Overall, cooking temperature, cooking time, dosage of alkali, oxygen pressure, and MgSO_4_ charge, *etc.* are the main factors influencing the results of soda-oxygen pulping.

#### 2.2.1. Effect of Temperature

Our attempt was to lower the cooking temperature while trying to maintain or even improve the properties of the obtained pulp. According to previous reports, the cooking temperature of soda-oxygen pulping for bagasse is usually not lower than 125 °C [21]. One possible reason for this is the concern that pulping below 125 °C could lead to low (screened) yields of well-separated pulp fibers. After an evaluation of the preliminary results from soda-oxygen pulping of bagasse under various temperatures (Table 2), it was found that soda-oxygen pulping of bagasse in a wide range of temperature (125 °C to 95 °C) could produce bagasse pulps with acceptable qualities. The screened yields of the obtained pulp reached *ca.* 60%, while the brightness was close to 60% or higher. Compared with results from previous studies, the current results are the best reported so far concerning soda-oxygen pulping of bagasse, implying that soda-oxygen pulping of the bagasse at low temperature (around 100 °C) is feasible. Both screened yield and brightness of the obtained pulps were not significantly changed with cooking temperature (from 125 °C to 105 °C). However, when cooking temperature was lowered to 100 °C or 95 °C, the brightness of the resultant pulp rapidly dropped below 60%, being 56.3% or 55.6%, respectively.

**Table 2:** Effects of maximum pulping temperature on soda-oxygen pulping of the bagasse.

T_max_ (°C)	Pulp Properties					Black Liquor	
	Screened Yield (%)	Rejects (%)	Kappa Number	Viscosity (dm^3^/kg)	Brightness (% ISO)	pH Value	Residual NaOH (g/L)
125	59.14	0.35	8.9	721	60.8	9.42	0.24
120	59.11	0.47	9.9	734	64.2	9.90	0.30
115	59.14	0.44	11.2	739	65.2	10.48	0.66
110	60.42	0.37	12.4	745	64.6	11.76	1.66
105	60.89	0.53	14.3	763	62.7	12.22	2.32
100	60.73	1.82	18.0	809	56.3	12.55	4.32
95	58.96	3.96	18.3	815	55.6	12.65	5.83

Active alkali charge 23%, time to maximum temperature 55 min, time at maximum temperature 90 min, initial pressure of oxygen 0.6 MPa, MgSO_4_ charge 0.5%, and de-pithed bagasse consistency 12%.

In addition the cooking temperature also affected the Kappa number of the pulp and pH values of the black liquor following the cooking. When pulping at higher temperatures, the more severe conditions resulted in a faster delignification and carbohydrate degradation of the bagasse, while consumption of alkali was accelerated leading to lower pH values of the black liquor. As a result, pulping at 125 °C gave the lowest Kappa number (8.9) and viscosity (721 dm^3^/kg) of the pulp, and also lowest residual alkali (0.24 g/L) of the black liquor. In contrast, pulping at lower temperatures would slow down the pulping reaction due to the milder conditions. The properties of resultant pulp accordingly showed an opposite change to that at higher temperature. The brightness of the pulp produced at 125 °C cooking temperature was 61% ISO, lower than that of pulp obtained at 120–105 °C, which was probably due to the low pH value (below 9.5) of the black liquor at the end of pulping. The shortage of alkali would inevitably depress the oxidative bleaching in soda-oxygen pulping. However, the brightness of the pulps produced at 100 °C and 95 °C was the lowest because of the insufficient delignification. The Kappa numbers of the pulps obtained at 100 °C and 95 °C, were over 18.0 in accordance with the brightness. Therefore, the lowest screened yield and highest rejection of pulps obtained at 95 °C, suggesting that pulping conditions at or below 100 °C were too weak for the soda-oxygen pulping to produce bagasse pulps with acceptable properties.

Listed in Table 3 are the properties of bagasse pulps produced by soda or soda-AQ pulping, and pulps following an oxygen bleaching (delignification). Comparing the data listed in Table 2 and Table 3, it is found that soda-oxygen pulping of bagasse has distinct advantages over soda or soda-AQ pulping. In particular the brightness of the pulps obtained at around 110 °C by soda-oxygen pulping were much higher than oxygen delignified soda or soda-AQ pulp. Compared with the pulps produced from other pulping methods, the properties of soda-oxygen pulp obtained at around 110 °C showed notable advantages, especially for the yields and brightness [4,22,23]. Taking all factors into consideration, there is a potential for soda-oxygen pulping at around 110 °C to produce bagasse pulps with screened yield up to 60%, Kappa number around 12, brightness close to 65%, and viscosity of 750 dm^3^/kg.

**Table 3:** Properties of soda and soda-AQ brownstock pulps, and their oxygen delignified pulps [19].

Pulping Method and Pulps		Screened Yield (%)	Rejects (%)	Kappa Number	Viscosity (dm^3^/kg)	Brightness (% ISO)
Soda	Brownstock	50.1	3.6	21.5	900	35.6
	Oxygen Delignified	48.4	-	11.8	820	45.0
Soda-AQ	Brownstock	49.3	1.2	13.3	860	37.2
	Oxygen Delignified	48.1	-	6.8	790	49.9

(1) Pulping process conditions: alkali charge 12%, cooking temperature 165 °C, time to 100 °C 30 min, time to maximum temperature 60 min, time at maximum temperature 60 min, AQ charge 0.1%, and bath ratio 1:5; (2) Oxygen delignification conditions: alkali charge 2.5%, MgSO_4_ charge 1.0%, temperature at time of O_2_ discharge 55 °C, maximum temperature 115 °C, time to maximum temperature 30 min, time at maximum temperature 60 min, oxygen dosage 5.0 kg/cm^2^, and consistency 10%.

#### 2.2.2. Effects of Cooking Time

As discussed above, the pulping at or below 100 °C was not enough to produce bagasse pulps with acceptable properties. However, pulping at lower temperature means less energy is needed, less carbohydrates are degraded and simpler operation, therefore pulping at 100 °C with various cooking times was tried in order to find suitable conditions for soda-oxygen pulping process.

Under the conditions shown in Table 4, it was found that when the cooking time was extended from 90 min to 180 min, the Kappa number and the viscosity of the pulp decreased accordingly from 18 to 14 and from 809 to 766 dm^3^/kg, respectively whereas the brightness of the pulp increased from 56.3% to 63.7% ISO, so the decrease in Kappa number was in accordance with the increasing brightness of the resultant pulp as the cooking time was extended. Moreover, much lower Kappa numbers and higher brightness of the pulp produced under these conditions means significantly less chemicals will be needed during bleaching, which is both economically and environmentally beneficial [19]. However, a significant reduction in the viscosity (from 806 to 766 dm^3^/kg) was found when the cooking time was prolonged from 120 to 180 min. There was no significant change in screened yield with the extended cooking time, which means most of the carbohydrates were still preserved even the degree of polymerization of cellulose decreased. This might be due to the relative mild cooking conditions used in this work. In addition, the decrease of rejects suggests more bagasse was converted into pulp fiber as the cooking time was increased, which also implies prolonging the cook time could improve the pulping results.

**Table 4:** Effects of cooking time at 100 °C on soda-oxygen pulping of the bagasse.

Cooking Time (min)	Pulp Properties					Black Liquor	
	Screened Yield (%)	Rejects (%)	Kappa Number	Viscosity (dm^3^/kg)	Brightness (%ISO)	pH Value	Residual NaOH (g/L)
90	60.73	1.82	18.0	809	56.3	12.55	4.32
120	61.07	1.18	16.4	806	61.3	12.48	3.96
180	60.93	0.51	14.0	766	63.7	11.94	2.19

All the pulping were carried out in the same condition as in Table 2 except the cooking time.

In general the extending cooking time significantly increased the delignification of bagasse in the soda oxygen pulping process, while it inevitably resulted in some degradation of the carbohydrates. Meanwhile, the extending cooking time would increase the energy cost, so cooking times longer than 180 min were not investigated in this study. As the results showed, the highest brightness of 63.7% ISO with a screened yield of 60.93% and Kappa number 14.0 was achieved by extending the cooking time to 180 min. Therefore, a longer cooking time was preferable when soda-oxygen pulping at 100 °C with cooking times from 90 min to 180 min. The obtained pulp was even better than that obtained at 105 °C cooking temperature (see also Table 2) in terms of the Kappa number, brightness and rejects of pulp obtained. In spite of the relative lower viscosity (still acceptable) of pulps produced, the suitable cooking time for soda-oxygen pulping at 100 °C was determined to be 180 min.

#### 2.2.3. Effect of Alkali Charge

It is well known that oxygen delignification is usually carried out in an alkaline medium, so alkali charge is an important factor for soda-oxygen pulping [24]. Trials have been done to evaluate the effects of alkali charge on pulping. Based on the forgoing experiment, the dosage of NaOH was set to be 22%, 23%, and 24% (wt % on raw materials), respectively, under the conditions shown in Table 5. As the results showed, the screened yields, the Kappa numbers and the brightness of pulps obtained under these conditions were very close, implying that the increase of alkali charges from 22% to 24% could, beyond expectation, not impact the pulping results to any significant degree although there were slightly differences over the range of alkali charges. These discrepancies may be caused by the inhomogeneity of the bagasse used here. The viscosity of the obtained pulps reduced with the increase in alkali charge more significantly than other properties (e.g., Kappa number, brightness and yield) because the cellulose degraded fast, supporting the general idea that delignification selectivity decreases with the increasing alkali concentration. Overall, the alkali charge of 23% could be the best choice for soda-oxygen pulping under the conditions used here.

**Table 5:** Effects of alkali charge on soda-oxygen pulping of the bagasse.

Dosage of NaOH (%)	Pulp Properties					Black Liquor	
	Screened Yield (%)	Rejects (%)	Kappa Number	Viscosity (dm^3^/kg)	Brightness (%ISO)	pH Value	Residual NaOH (g/L)
22	60.85	0.97	14.7	786	63.2	11.57	1.73
23	60.93	0.51	14.0	766	63.7	11.94	2.19
24	60.25	0.33	13.9	743	63.9	12.16	3.01

All the pulping were carried out in the same condition as in Table 4 except the alkali charge, and the cooking time was 180 min.

#### 2.2.4. Effect of Initial Pressure of Oxygen

The initial pressure of oxygen was used as a measure of oxygen dosage since soda-oxygen pulping is usually carried out in a sealed reactor of a certain volume. As mentioned earlier, the delignification of bagasse is a synergistic reaction of alkali and oxygen in the multiple phases of the gas–liquid–solid pulping system. The oxygen usage (pressure) could significantly influence the delignification of bagasse during cooking. In order to better understand the influence of the initial oxygen pressure on delignification, cooking trials were carried out under the following conditions: active alkali charge 23%, time to maximum temperature 55–65 min, time at maximum temperature 180 min, MgSO_4_ charge 0.5%, and de-pithed bagasse consistency 12%, and the initial pressures of oxygen were set as 0.4, 0.5, 0.6 and 0.8 MPa, respectively.

**Figure 1:**
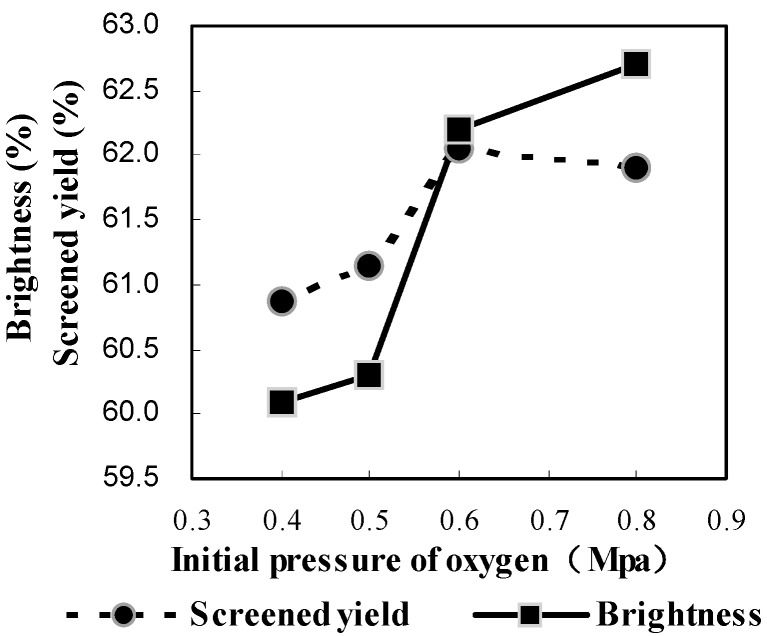
Influence of initial pressure of oxygen on screened yield and brightness.

Figure 1 indicates that the brightness increased (from 60.1% to 62.7% ISO) along with the increase of initial oxygen pressure from 0.4 to 0.8 MPa, whereas the screened yield increased significantly first (from 0.4 to 0.6 MPa) and then dropped slightly (from 0.6 to 0.8 MPa). These results indicated that the cooking condition become much hasher as the initial oxygen pressure increased from 0.6 to 0.8 MPa resulting in more degradation of cellulose at 0.8 MPa.

**Figure 2:**
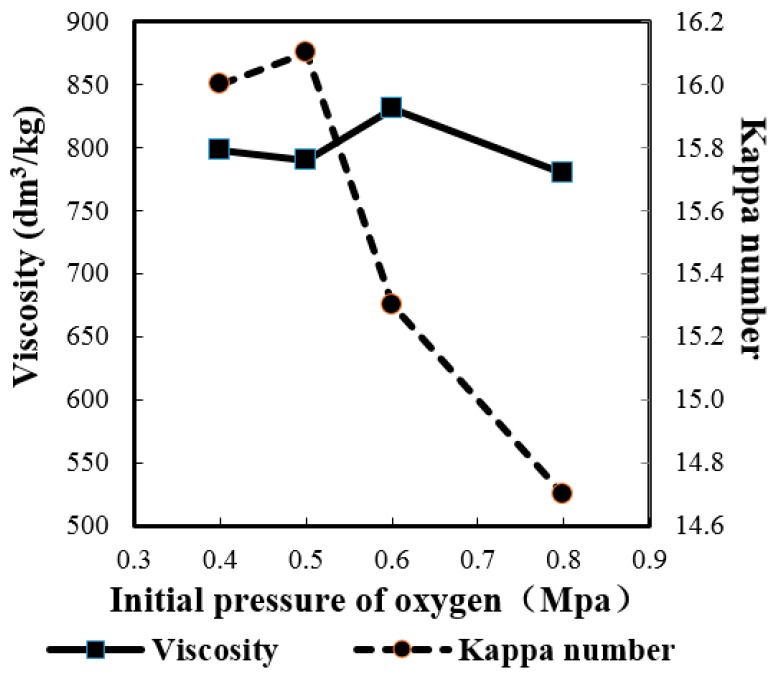
Influence of initial pressure of oxygen on viscosity and Kappa number.

In another words, the selectivity of delignification decreased as the initial oxygen was varied from 0.6 to 0.8 MPa. Theoretically the Kappa number and viscosity of pulp should decrease as the initial pressure was increased. However, the viscosity showed an unexpected maximum at 0.6 MPa (830 dm^3^/kg), although other viscosity data had a downward trend as the increasing of initial oxygen pressure (Figure 2). On the contrary, the Kappa number of the obtained pulp, as expected, decreased as the initial oxygen pressure increased from 0.4 to 0.8 MPa (Figure 2) except for the initial pressure of 0.5 MPa. Meanwhile, when the initial oxygen pressure changed from 0.4 to 0.5 MPa, the Kappa number, screened yield, brightness and viscosity almost did not change, which means that such a change in the initial oxygen pressure was not enough to cause any observable change in the resultant pulp. The results obtained from the pulping performed under various initial oxygen pressures showed inconsistencies with those from previous work, for example, slightly lower brightness companied with relative higher Kappa number and viscosity. This discrepancy may be caused by the inhomogeneity of the bagasse (such as incomplete de-pithing) as mentioned above, but the overall tendency of the results was consistent with the expected ones. Taking all this into account, a suitable initial oxygen pressure would be 0.6 MPa.

#### 2.2.5. Effect of MgSO_4_ Charge

The most common additive for oxygen delignification (bleaching) is MgSO_4_ which is used to protect carbohydrates from degradation. The presence of magnesium-containing compounds in the oxygen delignification process could be beneficial for pulp quality under certain conditions, and the mechanism for improving the selectivity of oxygen delignification by addition of magnesium is still not certain. However, the magnesium hydroxide precipitate formed under alkaline conditions certainly plays important roles, including forming complexes with transition metals and interacting with the carboxylic acid groups of carbohydrates to minimize oxidative degradation of carbohydrates [25]. In this work, MgSO_4_ was used as protective agent in the soda-oxygen pulping and its effect on the pulp properties was investigated.

**Figure 3:**
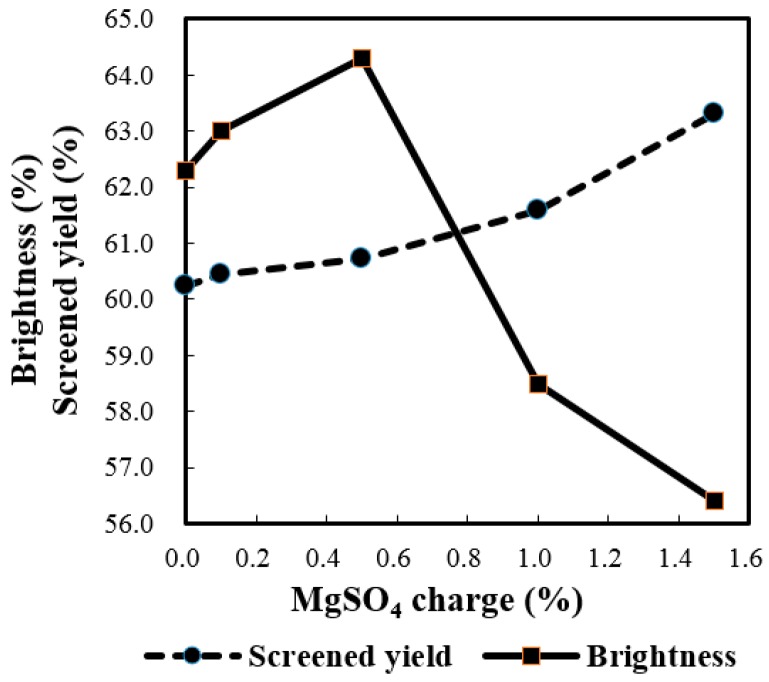
Influence of MgSO_4_ charge on screened yield and brightness (active alkali charge 23%, time to maximum temperature 55–65 min, time at maximum temperature 180 min, initial pressure of oxygen 0.6 MPa, and de-pithed bagasse consistency 12%).

**Figure 4:**
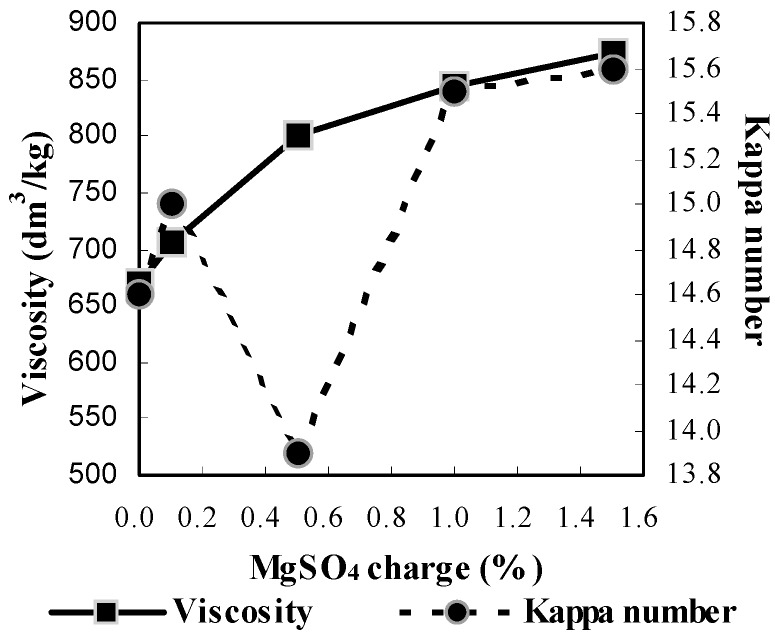
Influence of MgSO_4_ charge on viscosity and Kappa number (the same cooking conditions with Figure 3).

Figure 3 and Figure 4 illustrate the influence of MgSO_4_ charges from 0.0% to 1.5% on the resultant pulps. As the results show, the overall tendency of the screened yield, viscosity, as well as Kappa number increased steadily as the MgSO_4_ increased, except for the Kappa number at a MgSO_4_ charge of 0.5%. Both the screened yield and the intrinsic viscosity have been improved, although the Kappa number didn’t show much change (around 15.0). This indicated that the increasing addition of MgSO_4_ within a certain range could give rise to a better protection of carbohydrates during delignification. Meanwhile, the brightness increased first and then dropped sharply as the MgSO_4_ charge increased from 0.0% to 1.5%, and the maximum value of 64.3% ISO was achieved at 0.5% MgSO_4_ charge. Higher than 0.5% charge of MgSO_4_ inhibited the delignification resulting in a decrease in the brightness of the pulp. Although the screened yield and viscosity reached the highest level (63.31% and 873 dm^3^/kg, respectively) at 1.5% charge of MgSO_4_, the corresponding brightness of 54.6% ISO was the lowest. Therefore, the 0.5% MgSO_4_ charge was the best choice under conditions used.

#### 2.2.6. Effect of Bagasse Consistency

**Figure 5:**
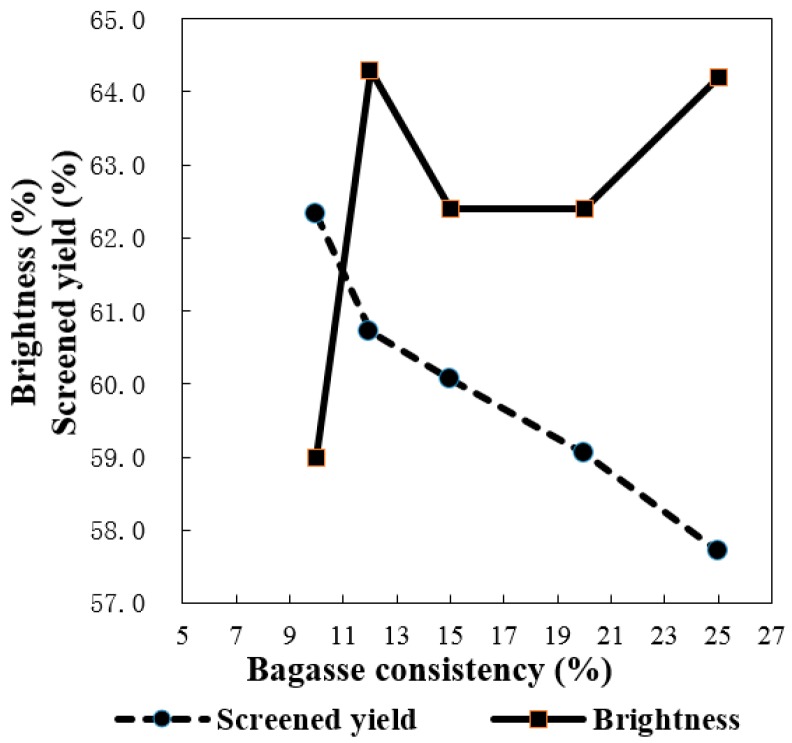
Effect of bagasse consistency on screened yield and brightness (active alkali charge 23%, time to maximum temperature 55–65 min, time at T_max_ 180 min, initial pressure of oxygen 0.6 MPa, and MgSO_4_ charge 0.5%, while the bagasse consistency varied as 10%, 12%, 15%, 20%, and 25%, respectively).

**Figure 6:**
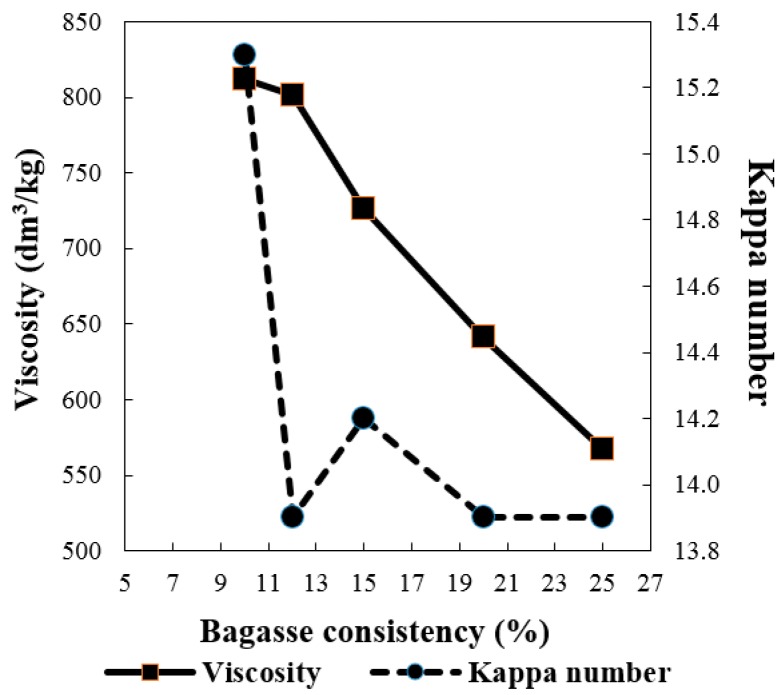
Effect of bagasse consistency on viscosity and Kappa number (same cooking conditions with Figure 5).

Sufficient cooking liquid is required to ensure a better or complete soaking of raw material with chemicals during the pulping process so that quality pulp is produced. Therefore the bagasse consistency, namely its solid-to-liquid ratio, is an important factor being evaluated here. In this study, various bagasse consistencies (*i.e.*, 10%, 12%, 15%, 20%, and 25%) were used for soda oxygen pulping. The results are shown in Figure 5 and Figure 6. When the bagasse consistency increased from 10% to 25%, both the screened yield and viscosity dropped significantly, *i.e.*, from 62.33% to 57.71% and 812 to 567 dm^3^/kg, respectively. It is expected that cooking with high consistency bagasse will reduce the energy and water consumption for pulping. However, the higher of the consistency means a stronger alkalinity when the same alkali charge is used, which could lead to more severe cellulose degradation. This was why the sharp decline in screened yield and viscosity was observed as the bagasse consistency increased. The lowest brightness of pulp occurred at 10% of bagasse consistency because the lowest alkalinity of cooking liquid was not strong enough to delignify the bagasse resulting in the highest Kappa number of the pulp. However, when the consistency varied from 12% to 25% the Kappa number of pulps became stable around 14.0, so a bagasse consistency of 12% can be considered the best for oxygen pulping under the current conditions in terms of the overall quality of the resultant pulp.

The goal of pulping is to remove as much lignin as possible from the bagasse, while retaining as much carbohydrates as possible. Based on the results we discuss above (Figure 1, Figure 2, Figure 3, Figure 4, Figure 5 and Figure 6), it was possible to produce quality bagasse pulps with Kappa number around 14, screened yield of 60% and brightness of 60% ISO by the soda-oxygen pulping process under the following conditions: initial oxygen pressure of 0.6 MPa, NaOH charge of 23%, MgSO_4_ charge of 0.5%, bagasse consistency of 12%, cooking temperature of 100 °C with a cooking time of 180 min, and cooking temperature of 105 °C or 110 °C with a cooking time of 90 min, respectively. Overall, the soda-oxygen pulping process at 100 °C with a longer cooking time of 180 min produced pulp with better viscosity, while pulping at 110 °C with a shorter cooking time of 90 min showed better delignification as the obtained pulp presented relative lower Kappa number and higher brightness.

## 3. Experimental Section

### 3.1. Materials

The bagasse used in this study was obtained from Yunnan Xinping Nan’en Sugar and Paper Co., Ltd. (Xinping, Yunnan Province, China). The bagasse was screened, de-pithed and air-dried. All the chemicals were analytical grade and used as supplied.

### 3.2. Chemical Composition Analyses

A portion of de-pithed bagasse was ground and the 40–60 mesh fraction was used for chemical analysis. The analysis of typical composition as follows: cellulose (Kurschner–Hoffner method), Holocellulose (Chlorination method) and lignin (TAPPI T 222 om-88), 1% NaOH extractives (TAPPI T 212), pentosan (TAPPI T 223 cm-01).

### 3.3. Pulping Process

Soda-oxygen pulping of bagasse was carried out in 1 Liter (×4) stainless steel reactors set up in a 15-L thermoelectrical rotating autoclave. The parameters for pulping were maximum cooking temperature 125 to 95 °C, cooking time 90 min, 120 min, and 180 min, alkali charge 22%, 23%, and 24%, initial pressure of oxygen 0.4, 0.5, 0.6 and 0.8 MPa, MgSO_4_ charge 0%–1.5%, and de-pithed bagasse consistency 10%, 12%, 15%, 20%, and 25%, *etc.* The resultant pulp was thoroughly washed with water and screened.

### 3.4. Analysis of Pulp Properties

Pulp yield was determined gravimetrically following drying at 105 ± 2 °C for 24 h. Properties of pulp such as Kappa number (ISO 302:1981, Reapproved 1991), viscosity (ISO 5351-1:1981, Reapproved 1991), and brightness (ISO 3688:1999, ISO 2470: 1999), were measured by the referenced standard methods.

## 4. Conclusions

Quality bagasse pulp with satisfactory properties has been produced by a soda-oxygen pulping process performed at 100 °C, the lowest temperature reported so far for chemical pulping. Similar pulps were obtained using three different sets of conditions: alkali charge 23%, initial pressure of oxygen 0.6 MPa, MgSO_4_ charge 0.5%, and de-pithed bagasse consistency 12%, T_max_ 100 °C with cooking time at T_max_ 180 min, and T_max_ 105 °C or 110 °C with cooking time at T_max_ 90 min, respectively. The resultant pulps produced under such conditions had screened yields up to 60%, brightness close to 65% ISO, viscosity near 800 dm^3^/kg, and Kappa numbers around 13. The production of bagasse pulp with screened yield and brightness exceeding 60% by soda-oxygen pulping at temperature down to 100 °C is unprecedented and would make great contribution to the development of environmental friendly, energy-saving, and clean pulping processes for non-wood raw materials.
